# A Case Report on a Common Tumour With an Uncommon Presentation: Glioblastoma

**DOI:** 10.7759/cureus.66830

**Published:** 2024-08-14

**Authors:** Chathuri L Munagama, Varithamby Rajendiran, Shehan Silva

**Affiliations:** 1 University Medical Unit, Colombo South Teaching Hospital, Colombo, LKA; 2 Neurology, Colombo South Teaching Hospital, Colombo, LKA; 3 Medicine, Colombo South Teaching Hospital, Colombo, LKA; 4 Medicine, Faculty of Medical Sciences, University of Sri Jayewardenepura, Boralesgamuwa, LKA

**Keywords:** neuropsychiatric manifestation, cns malignancy, brain biopsy, glioblastoma, brain tumour

## Abstract

The most common type of primary brain tumour in adults is gliomas although rare. Glioblastomas are a subtype of gliomas with the worst prognosis having the ability to rapidly increase in size, even doubling within days to weeks. Patients can present with varied presentations depending on the site of the involvement, thus misleading in diagnosis due to vagueness. The most common clinical presentations include headaches, seizures, and focal neurological signs. However, there can be atypical presentations like personality changes and back pain due to meningeal irritation which may be the only presenting complaint in the early stages. Magnetic resonance imaging (MRI) is usually considered the only investigation required for the diagnosis of the illness. However, it can mislead in the early stages. Therefore, brain biopsy remains the gold standard in the diagnosis of glioblastoma multiforme. It is important to identify the subtype to decide on the prognosis and plan the management thereafter. Here, we present a 49-year-old woman with prominent personality changes, depressive symptoms, and atypical brain imaging findings. The definitive diagnosis was made with the brain biopsy as two MRI findings were contradictory. This article highlights the importance of suspicion of primary brain tumours in adults presenting with atypical neuropsychiatric manifestations.

## Introduction

Adult-type diffuse gliomas are primary brain tumours which are classified by the World Health Organization (WHO) as grade IV [1]. This designation is assigned to cytologically malignant, mitotically active, necrosis-prone neoplasms typically associated with fatal outcomes. The five-year survival rate for patients with glioblastoma is estimated to be only 6.9%, while the average period is estimated to be only eight months [1]. Headaches, vomiting, and seizures are the most common presentations [2]. However, in some patients, the initial presentation may mislead due to atypical clinical presentations like psychotic symptoms, personality changes, mood disturbances, behavioural disorders, and visual and auditory hallucinations [3,4]. It is therefore important to always exclude an organic brain disorder in patients presenting with psychiatric manifestations.

Magnetic resonance imaging (MRI) may not demonstrate a typical mass leading to a difficult diagnosis with the differentials including infectious, inflammatory, autoimmune, or vasculitic disease. Typical features of a demyelinating process might not be present at the very early stages. Hence, glioblastoma should still be considered one of the differential diagnoses in a middle-aged patient with an unexplained clinical presentation who does not respond to the conventional treatment for suspected medical or psychiatric illness [5].

## Case presentation

A 49-year-old woman presented with a history of behavioural disturbances, walking difficulty, visual disturbances, and memory impairment gradually worsening over two months. She had a severe generalized headache over this period. A non-contrast computed tomography (NCCT) done at that time was normal. There were recurrent staring episodes, and her consciousness level changed from time to time. The patient was treated for depression by the primary care physician. She could not recall these staring episodes. She had no documented fever before admission although there was a fever spike as in-patient. There was no significant past medical history including malignancy and no close contact history of tuberculosis. There was no history of alcohol or narcotic usage and a significant travel history.

She was afebrile with a pulse rate of 72/min and a blood pressure of 130/80 mmHg. The Glasgow Coma Score was 13/15 (eye: 4, motor: 5, verbal: 4). The complete neurological examination was normal initially other than confusion with regard to time, place, and person. The rest of the systems examination including breasts were normal. Both optic discs and maculae were unremarkable.

An ill-defined hypodense area in the left parietal area with mild cerebral oedema and midline shift to the contralateral side was evident in the NCCT of the brain compared to the previous NCCT. Focal cerebritis and a space-occupying lesion were considered as differential diagnoses. MRI showed intracranial appearance with vasogenic oedema and cytotoxic oedema of the splenium, the body of the corpus callosum, and the limbic system including the hippocampus and the insular cortex consistent with herpes encephalitis. Intravenous (IV) acyclovir and IV dexamethasone were also added for the initial 48 hours. An electroencephalogram (EEG) performed for persistent staring episodes reported a moderate degree of encephalopathy with episodic spike and spike-wave in the anterior temporal region indicating severe electrical seizure activity. Antiepileptics commenced resulting in the improvement of staring episodes. However, this was followed by visual blurring and weakness of the right hand. The fever continued to linger despite IV antiviral therapy. The investigations are depicted in Table 1 and Table 2.

**Table 1:** Basic investigations CRP: C-reactive protein; CSF: cerebrospinal fluid

Investigation	Patient's value	Reference range
White blood count	7,910	4,000-11,000 µL
Neutrophils	82.5%	50-75%
Lymphocytes	10.1%	20-40%
Haemoglobin	12.1	12-16 g/dL
Platelet	296,000	150,000-450,000 µ/L
Aspartate transaminase	43.5	<50 U/L
Alanine transaminase	46.6	<50 U/L
Alkaline phosphatase	114	47-140 U/L
Albumin	36.1	35-52 g/L
Globulin	38.57	23-34 g/L
Total bilirubin	11.6	5-21 µmol/L
Direct bilirubin	5.1	<3.4 µmol/L
Indirect bilirubin	6.5	3.4-12 µmol/L
Creatine phosphokinase	12.8	10-120 µg/L
Serum magnesium	0.8	0.85-1.10 mmol/L
Serum albumin corrected calcium	2.4	2.02-2.6 mmol/L
Serum creatinine	79.7	74-110 µmol/L
Serum sodium	139	135-145 mmol/L
Serum potassium	3.8	3.5-4.5 mmol/L
Erythrocyte sedimentation rate	75	mm/1st hour
CRP	<5	<5 mg/dL
CSF protein	45.1	15-60 mg/L
CSF polymorphs	Nil	0
CSF lymphocytes	Nil	0-5 WBC/mm^3^
CSF culture	No growth	<3 WBC/mm^3^

**Table 2:** Other investigations CSF: cerebrospinal fluid; NMDAR: N-methyl-D-aspartate receptor; CASPR2: contactin-associated protein-like 2: AMP AR-1/2: activated protein kinase alpha subunit; LGL-1: leucine-rich glioma-inactivated 1; DPPX: dipeptidyl peptidase-like protein-6; GABARB-1/2: gamma-aminobutyric acid receptor B; NCCT: non-contrast computed tomography; EEG: electroencephalogram; MRI: magnetic resonance imaging; CECT: contrast-enhanced computed tomography

Investigation	Comment
CSF cytology	No inflammatory or malignant cells noted
Venereal disease research laboratory test	Negative
Retroviral study	Negative
Urinalysis	Normal
Chest X-ray	Normal
NMDAR antibody	Negative
CASPR2 antibody	Negative
AMP AR-1/2 antibody	Negative
LGI-1 antibody	Negative
DPPX antibody	Negative
GABARB-1/2 antibody	Negative
Blood picture	Moderate rouleaux formation and left shift of white blood cells with neutrophil predominance and occasional myelocytes. Can be infective, inflammatory, and neoplastic pathology
NCCT brain	An ill-defined hypodense area in the L/S parietal region with mild cerebral oedema and midline shift to the contralateral side. Possible focal cerebritis or space-occupying lesion
First EEG	Encephalopathic seizure activity
Second EEG after one week of the first EEG	Compared to the previous, there's a marked improvement in the encephalopathic seizures
First MRI of the brain on the day of admission	Intracranial appearance with vasogenic oedema as described above and cytotoxic oedema of the splenium and the body of corpus callosum are consistent with herpes encephalitis
Second MRI of the brain on the 14th day	Areas of vasogenic oedema involving the inferior aspect of bilateral frontal lobes, cerebral peduncles extending down to the lateral aspect of the brainstem, and corpus callosum with areas of nodular contrast enhancement in the left side periventricular region and splenium of the corpus callosum. Magnetic resonance spectrometry indicating tissue necrosis. Thicken and contrast enhancement of optic chiasm and intracranial segments of bilateral optic nerves extending to optic radiation near chiasm. Possibilities such as severe viral encephalitis with tissue necrosis, atypical infective meningoencephalitis, central nervous system lymphoma, demyelinating illness, and bacterial infection are less likely
Third MRI of the brain on the 20th day	Multifocal lesions involving the splenium of the corpus callosum and left cerebral hemisphere as described with associated white matter oedema and mass effect demonstrating diffusion restriction and heterogenous peripheral contrast enhancement are favoured to be neoplastic in origin rather than inflammatory
CECT of the chest, abdomen, and pelvis	Normal
Bilateral mammography	Normal

There were no metabolic derangements. The blood picture did not show evidence of a haematological malignancy such as lymphoma. Microbiological studies of the cerebrospinal fluid (CSF) were negative for infections, while the cytology and autoantibody profile too were negative. Contrast-enhanced CT (CECT) of the chest, abdomen, and pelvis as well as bilateral mammography did not demonstrate any malignancy, thus excluding metastatic disease of the brain. Despite optimum management for the probable diagnosis of central nervous system (CNS) infection, the patient deteriorated.

**Figure 1:**
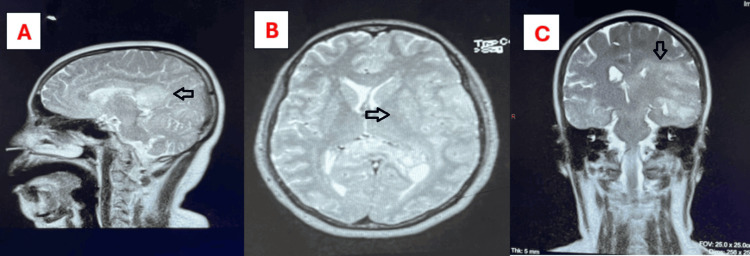
(A) Sagittal view, (B) axial view, and (C) coronal view of the T2 images of the last MRI of the brain and arrows showing multifocal lesions involving the splenium of the corpus callosum and the left cerebral hemisphere as described with associated white matter oedema and mass effect demonstrating diffusion restriction and heterogenous peripheral contrast enhancement MRI: magnetic resonance imaging

She underwent a brain biopsy upon findings of the second MRI which showed changes in tissue necrosis and vasogenic oedema with a radiological diagnosis of severe viral encephalitis with tissue necrosis, and the third MRI showed neoplastic process. Samples were taken from multiple sites of the brain following site marking (Figure 1).

**Figure 2:**
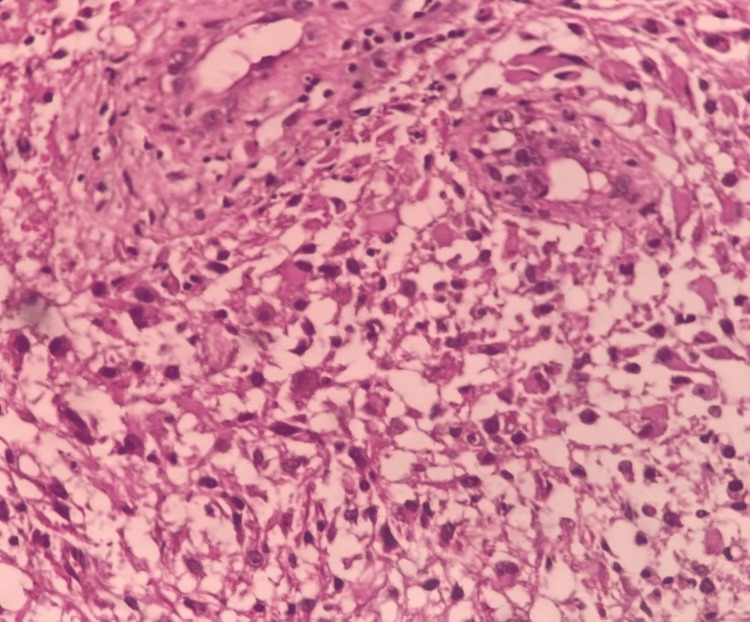
The brain biopsy showing highly cellular glial tumour with atypia demonstrating microvascular proliferation with increased mitotic activity and necrosis

The biopsy showed multifocal lesions involving the corpus callosum, ventricles, and hippocampal areas, and the conclusion was adult-type diffuse glioma CNS WHO grade IV. Immunohistochemistry was not done due to the unavailability of the test. The patient's condition rapidly deteriorated; she succumbed within one month of the diagnosis despite the chemoradiation (Figure 2).

## Discussion

Histological typing of tumours of the nervous system was first described by Zülch in 1979. The same consensus is used today with some modifications [6]. Gliomas are the most prevalent type of adult brain tumour, accounting for 80% of malignant brain tumours arising from supporting cells, glia [7]. Patients diagnosed to have low-grade glioma (WHO grade II) have better survival of 5-15 years, whereas, for grade III tumour, it is 2-3 years. In patients with WHO classification of adult-type diffuse gliomas grade IV tumours (also known as glioblastoma multiforme (GBM)), the median survival does not exceed 12-14 months [8]. 

The usual presentation of brain tumours varies from the site of involved brain tissues. Common symptoms include headache, seizures, focal neurologic symptoms like memory impairment, motor weakness, visual disturbances, language deficit, and cognitive and personality disorders [1]. Following diagnosis, patients may experience psychological affliction and affective disorders. Manic states, anxiety, low mood with depression, and even suicidal ideation can occur. Furthermore, shock and unsettling and intrusive thoughts about the disease may be prominent. Often, these emotional reactions are mild but sometimes according to the severity and/or persistence suggest an adjustment disorder or a major depressive disorder [9].

In the absence of common presentations, some may present with neuropsychiatric symptoms as the initial symptoms, before the diagnosis which can be rare. Subtle and atypical neuropsychiatric symptoms may predominate early during tumour growth/expansion. Later, they can present with compressive symptoms when the tumour gets bigger. Psychiatric manifestations related to glioblastoma in the cases retrieved here have been reported in adults whose ages spanned 24-65 years; most (62%) of the affected patients were middle-aged [9]. 

Even though the outcome is poor, the diagnosis must be made early so as to improve the quality of life, initiating therapeutic management and psychological management even palliation. However, this atypical presentation can mislead medical professionals and can delay clinical suspicion and diagnosis [3,10].

As imaging studies, brain MRI with contrast is often the only study required before the surgery. Patients with a contraindication to brain MRI should undergo brain CT with contrast. Screening for systemic malignancy is not compulsory when the clinical and radiographic suspicion for high-grade glioma is high [1].

Glioblastoma is typically recognized as an iso- or hypointense lesion on T1- and a hyperintense lesion on T2-weighted MRI scans. Moreover, high-grade gliomas such as glioblastoma are often visualized as enhancing lesions with central necrosis, while low-grade gliomas tend to show minimal contrast enhancement [11]. The presented case highlights the importance of histopathological examination and repeated imaging findings with clinical deterioration of the patient to establish the diagnosis of GBM. Although the patient's MRI initially suggested a non-enhancing lesion, raising the suspicion of an infective and inflammatory process, repeated MRI and brain biopsy were necessary to confirm the final diagnosis. Failure to obtain a biopsy may have resulted in misdiagnosis or delayed diagnosis and ineffective treatment for the patient. Therefore, tissue diagnosis without delay in a patient with a high-grade glioma is essential [1].

## Conclusions

Even though primary brain tumours are rare among adults, it is not uncommon to have atypical presentations. A low threshold should exist to exclude all the possible organic causes including cerebral tumour like glioma for the presentation of rapidly developing psychiatric symptoms especially in a patient who has been previously well. Imaging studies may be inconclusive to generate a definite diagnosis in some presentations. In such instances, histological means of diagnosis at the very early stages of the illness may be instrumental to prevent rapid progression and prevent complications such as mass effect. Furthermore, it would ensure the opportunity for early curative and/or palliative management in patients. So, they should proceed with histological diagnosis at the very early stages of the illness, to make future management decisions and to establish the diagnosis and prevent mass effect on surrounding structures, which can be rapidly fatal.
